# Incorporation of *Lippia citriodora* Microwave Extract into Total-Green Biogelatin-Phospholipid Vesicles to Improve Its Antioxidant Activity

**DOI:** 10.3390/nano10040765

**Published:** 2020-04-16

**Authors:** Francisco Javier Leyva-Jiménez, Maria Letizia Manca, Maria Manconi, Carla Caddeo, José Antonio Vázquez, Jesús Lozano-Sánchez, Elvira Escribano-Ferrer, David Arráez-Román, Antonio Segura-Carretero

**Affiliations:** 1 Functional Food Research and Development Center, Health Science Technological Park, Avenida del Conocimiento s/n, E-18016 Granada, Spain; jleyva@cidaf.es (F.J.L.-J.); darraez@ugr.es (D.A.-R.); ansegura@ugr.es (A.S.-C.); 2 Department of Scienze della Vita e dell’Ambiente, University of Cagliari, via Ospedale 72, 09124 Cagliari, Italy; mlmanca@unica.it (M.L.M.); manconi@unica.it (M.M.); caddeoc@unica.it (C.C.); 3 Group of Recycling and Valorization of Waste Materials (REVAL), Marine Research Institute (IIM-CSIC), C/Eduardo Cabello, 6, CP36208 Vigo, Spain; jvazquez@iim.csic.es; 4 Department of Food Science and Nutrition, University of Granada, Campus of Cartuja, 18011 Granada, Spain; 5 Biopharmaceutics and Pharmacokinetics Unit, Institute for Nanoscience and Nanotechnology, University of Barcelona, 08193 Barcelona, Spain; eescribano@ub.edu; 6 CIBER Physiopathology of Obesity and Nutrition (CIBEROBN), Institute of Health Carlos III, 28029 Madrid, Spain; 7 Department of Analytical Chemistry, Faculty of Sciences, University of Granada, Fuentenueva s/n, E-18071 Granada, Spain

**Keywords:** *Lippia citriodora*, green extract, phospholipid vesicles, antioxidant, *Thunnus albacares*, skin delivery

## Abstract

Phytochemicals from *Lippia citriodora* leaves were extracted by applying an innovative technology based on the use of microwaves, which represents an alternative method to extract bioactive substances. The obtained extract was incorporated into phospholipid vesicles in order to promote the antioxidant effect of the bioactive molecules present in *L. citriodora* extract. The extract was analyzed by High Performance Liquid Chromatography coupled to Time-Of-Flight mass spectrometer by electrospray (HPLC-ESI-TOF-MS) and different phytochemicals were detected and quantified. The whole extract was incorporated in liposomes, glycerosomes (liposomes modified with glycerol) and propylene glycol-containing vesicles (PG-PEVs). Moreover, a biopolymer obtained from fish by-product, that is *Thunnus albacares* skin, was added to improve the bioactivity of the formulations. The in vitro biocompatibility and the antioxidant efficacy of the extract in solution or loaded in the vesicles were tested in primary mouse embryonic fibroblasts (3T3). The results showed the superior bioactivity of the vesicle formulations over the aqueous solution of the extract, which points to an interesting strategy for the treatment of skin disorders.

## 1. Introduction

In recent decades, the role of inflammatory processes in the pathophysiology of age-related diseases and in the development of chronic degenerative diseases has gained interest in the scientific community. Inflammation is a complex biological response that starts in response to internal or external stimuli (e.g., metabolic dysfunctions or pathogens) and involves the upregulation of several enzymes and markers in damaged cells, such as reactive oxygen species (ROS) and reactive nitrogen species (RNS). Their accumulation in damaged cells and tissues, if extreme or prolonged, can lead to the development of several types of chronic diseases [1,2].

Additionally, even in the developed countries, the daily life modification has been associated with increased poor nutrition, consumption of poor-quality foods (in essential nutrients), insufficient physical activity and consumption of tobacco and alcohol. This dangerous lifestyle causes an overproduction of oxidants, thus an imbalance of oxidant and antioxidant levels in human tissues and an excessive accumulation of ROS. This condition is called oxidative stress and can damage cells, tissues and organs. It is involved in several age-related chronic diseases, such as cardiovascular, chronic obstructive pulmonary, neurodegenerative diseases and cancer, significantly affecting life expectancy [3].

Phytochemicals are being investigated as a natural alternative to treat skin conditions associated with inflammation and oxidative stress (e.g., dermatitis, psoriasis, or melanoma), due to their ability to counteract ROS and RNS. According to several studies, the topical administration of anti-inflammatory and antioxidant compounds of natural origin can ameliorate altered skin conditions and restore skin normal functions [4,5,6].

In this scenario, *Lippia citriodora*, also known as *Aloysia citriodora* or lemon verbena, has shown promising beneficial properties. It has been traditionally used as a beverage to calm fever, stomach-ache or gastrointestinal disorders [7]. Phenolic compounds represent a relevant part of the bioactive compounds of *L. citriodora*, with verbascoside and isoverbascoside being the major components [8]. They were proved to possess strong high antioxidant activity [9]. Flavonoids, such as chrysoeriol and luteolin derivatives, have also been detected in *L. citriodora* [10].

Despite the promising beneficial properties of phenolic compounds, their bioavailability in the skin can be hindered by poor solubility and stability [11]. Several strategies have been proposed to overcome these problems. For instance, liposomes and other innovative vesicular systems have been developed to enhance the skin delivery of phenolic compounds extracted from natural sources [12,13,14]. Previous studies underlined the key role played by glycols and polymers in affecting the ability of lamellar vesicles to carry natural molecules to the target sites [15]. The protective effects of phenolic compounds in phospholipid carriers against ROS has been widely demonstrated in vitro [16,17].

The aim of this work was to improve the bioavailability of *L. citriodora* extract by its incorporation into ad hoc formulated natural-based phospholipid vesicles. The extract was obtained by the ecosustainable microwave-assisted extraction, and the components were identified and quantified by HPLC-ESI-TOF/MS. The extract was incorporated in liposomes, glycerosomes (liposomes modified with glycerol) and propylene glycol-containing vesicles (PG-PEVs). Special attention was devoted to formulating phytoformulations by using natural, biocompatible components. Biopolymer-vesicles were also developed by adding a gelatin obtained from *Thunnus albacares* (a species of tuna). The physico-chemical properties of the vesicles and entrapment efficiency were assessed. The in vitro biocompatibility of the *L. citriodora* loaded in vesicles or in solution was evaluated in primary mouse embryonic fibroblasts (3T3), as well as their ability to protect the cells from oxidative stress.

## 2. Materials and Methods 

### 2.1. Reagents

Extraction procedures were performed by using a mixture of Milli-Q water (Millipore Bedford, MA, USA) and ethanol (VWR chemicals, Radnor, PA, USA). Analytical assays were performed by using acetonitrile and formic acid (Fisher chemicals, Waltham, MA, USA and Sigma-Aldrich, Steinheim, Germany, respectively). The calibration curves were built by using available commercial standards of verbascoside, loganic acid, kaempferol-3-glucoside, quercetin and apigenin purchased from Extrasynthese (Genay Cedex, France), Fluka or Sigma Aldrich (Steinheim, Germany). The vesicles were prepared by using soy phosphatidylcholine (Phospholipon^®^ 90G; P90G) purchased from Lipoid GmbH (Ludwigshafen, Germany). Glycerol and propylene glycol were purchased from Galeno (Carmignano, PO, Italy). Biogelatin was obtained from yellowfish tuna skin (*Thunnus albacares*) according to the processes previously described by Sousa et al. (2017). Briefly, a set of sequential treatments were applied (alkalis, mineral acid and organic acid) to prepare the fish skin for the thermal extraction (for 16 h at 45 °C) of the aqueous gelatin solution. Then, gelatin was purified by concentration and diafiltration using 30 kDa MWCO ultrafiltration membranes, and oven dried for 72 h at 50 °C. All steps were optimized in order to reduce, as much as possible, the concentration of reagents, the volume of water utilized and the time of processing [18].

### 2.2. Extraction and Phenolic Quantification by HPLC-ESI-TOF/MS

*L. citriodora* leaves, purchased from Monteloeder (Alicante, Spain), were milled by an ultra-centrifugal mill ZM200 (Retsch GmbH, Haan, Germany) to get an average particle size of 500 µm. The powder was stored in a dry and dark place until extraction. Microwave-assisted extraction (MAE) was performed in a Multiwave 3000SOLV (Anton Paar, Graz, Austria). An extract rich in polar compounds was obtained using 42% ethanol at 113 °C for 22 min [17]. Analytical assays were carried out by reverse-phase HPLC. The chromatographic separation was performed by using a RRLC 1200 series (Agilent Technologies, Palo Alto, CA, USA) with a Zorbax Eclipse Plus C18 analytical column (150 × 4.6 mm id, 1.8 µm particle diameter. Agilent Technologies, Palo Alto, CA, USA) and a multistep gradient elution with a mobile phase of water: acetonitrile 90:10 (v:v) with 0.1% formic acid (A) and acetonitrile (B) for 35 min.

The HPLC was coupled to a mass spectrometer equipped with an orthogonal electrospray interface (ESI; model G1607 from Agilent Technologies, Palo Alto, CA, USA) operating in negative ionization mode considering a mass range of 50–1000 m/z. An external calibration with sodium formate cluster was done in a quadratic high-precision calibration (HPC) regression mode. Source and transfer parameters were set according to a previously reported method [8].

The data provided by the analytical platform were processed by using DataAnalysis 4.0 software (Bruker Daltonics, Bremen, Germany), which allows the identification and quantification of the polar components of extracts. Hence, the qualitative characterization of each compound was made upon an interpretation of their mass spectra data provided by TOF-MS (m/z calculated and experimental, mSigma and molecular formula) and the information available in the literature [19]. For the quantitative characterization, four different calibration curves were built with verbascoside, loganic acid, quercetin and kaempferol-3-glucoside. The calibration curves were built by plotting the standard concentration as a function of the peak area given by HPLC-ESI-TOF analyses (area standard/area internal standard). Apigenin was used as internal standard (25 µg/mL). In all cases, the linearity of calibration curves was higher than 0.99. Detection and quantification limits, as well as repeatability of the method (intraday and interday precision) was previously reported [8].

The extract was dissolved in water:ethanol (50:50 v:v) to reach a concentration of 5 mg/mL. The concentration of the phytochemicals was calculated by interpolation of the area analyte/area internal standard ratio in the corresponding calibration curve. Iridoid glycosides were quantified using loganic acid calibration curve. According to their structures, luteolin-7-diglucuronide, chrysoeriol-7-diglucuronide, apigenin-7-diglucuronide and acacetin-7-diglucuronide were quantified by using the kaempferol-3-glucoside curve. The amounts of the rest of flavonoids were calculated by using the quercetin calibration curve. Lastly, verbascoside calibration curve was used to quantify the phenylpropanoids group. The results were expressed as mg of analyte per g of *L. citriodora* extract.

### 2.3. Vesicle Preparation

P90G (180 mg/mL) and the extract (50 mg/mL) were dispersed in water to prepare liposomes, and in a blend of glycerol or propylene glycol in water (25% *v*/*v*) to prepare glycerosomes and PG-PEVs, respectively. The composition of the vesicles is reported in Table 1. The dispersions were sonicated at an amplitude of 14 micron, 25 cycles, 5 seconds ON and 2 seconds OFF with a Soniprep 150 ultrasonic disintegrator (MSE Crowley, London, UK). After that, the formulations were stored in the dark at 4 °C until use.

### 2.4. Vesicle Characterization

The morphology of the vesicles was evaluated by cryogenic-transmission electron microscopy (cryo-TEM). The procedure followed to prepare the samples was previously reported by Manca et al. [15]. Briefly, a thin aqueous film was formed by placing a drop of each sample on a glow-discharged drilled carbon grid, and blotting the grid on filter paper. The film was then vitrified by plunging the grid (maintained at 100% humidity and room temperature) into ethane at melting point, by using a Vitrobot (FEI Company, Eindhoven, The Netherlands). After that, the vitreous film was transferred to a Tecnai F20 TEM (FEI Company, Eindhoven, The Netherlands) and the samples were observed in a low-dose mode. Image acquisition was made at 200 kV at a temperature between −170 and −175 °C with a CCD Eagle camera (FEI Company, Eindhoven, The Netherlands).

The physicochemical properties of the vesicles were checked by using a Zetasizer nano-ZS (Malvern Instruments, Worcestershire, UK). The average diameter (D) and polydispersity index (PDI) were determined by photon correlation spectroscopy, and the zeta potential by electrophoretic light scattering [20]. Prior to each analysis, samples were diluted (1:100) by using the appropriate mixture (water, water-glycerol or water-propylene glycol).

In order to evaluate the entrapment efficiency, the extract-loaded vesicles were purified from the non-entrapped bioactives by dialysis. Briefly, 1 mL of each formulation was loaded into dialysis tubes (Spectra/Por^®^ membranes, 12–14 kDa MW cut-off, Spectrum Laboratories Inc., Rancho Dominguez, CA, USA) and dialyzed against water for 2 h at 25 °C (refreshing water after 1 h). Both non-dialyzed and dialyzed samples were assayed with the DPPH (2,2-diphenyl-1-picrylhydrazyl) test. Each sample was diluted (1:50) with methanol and 60 µL of the dilution was added to a DPPH methanolic solution (40 μg/mL; 1940 µL). The samples were stored in the dark at room temperature for 30 min. After that, absorbance was measured at 517 nm. The antioxidant activity (AA) results were expressed as mg of gallic acid equivalents/mg of extract in the formulation. All experiments were performed in triplicate. The entrapment efficiency (EE) was calculated as the percentage of the antioxidant activity (AA) of dialyzed samples versus non-dialyzed ones, according to the following equation:

(1) $$EE\%=\frac{AA\;Dialyzed}{AA\;Non-dialyzed}\times 100$$

### 2.5. In Vitro Biocompatibility Assessment of the Formulations

To evaluate the biocompatibility of the vesicle formulations, primary mouse embryonic fibroblasts (3T3, ATCC collection, USA) were grown as a monolayer in 75 cm^2^ flasks, at 37 °C, 100% humidity and 5% CO_2_, using Dulbecco’s modified eagle’s medium (DMEM) with high glucose, 10% of bovine serum, penicillin-streptomycin and fungizone. 96-well plates were used to seed fibroblasts (2.5 × 10^6^ cells/well), which were incubated for 24 h. After that, the cells were treated for 48 h with the extract incorporated in the vesicles or dissolved in water, properly diluted to reach the required concentrations (50, 5, 0.5 and 0.05 µg/mL). The cells were washed with fresh medium and their viability was evaluated by the MTT (3(4,5-dimethylthiazolyl-2)-2, 5-diphenyltetrazolium bromide) colorimetric assay. One hundred microliters of the MTT reagent (0.5 mg/mL in PBS, final concentration) were added to each well. After 3 h, the formazan crystals produced in living cells, were dissolved in 100 µL of DMSO and the absorbance was read at 570 nm with a microplate reader (Synergy 4, Reader BioTek Instruments, AHSI S.P.A, Bernareggio, Italy). The experiments were made in accordance with the protocols described in ISO 10,993 [21] and at least in triplicate, and the results were expressed as a percentage of cell viability in comparison with untreated cells (100% viability).

### 2.6. Protective Efficacy of the Formulations against Cells Stressed with Hydrogen Peroxide

The ability of *L. citriodora* extract in solution or loaded in the vesicles to protect fibroblasts from oxidative stress was evaluated. The cells were seeded in a 96-well plate and incubated at 37 °C, 100% humidity and 5% CO_2_ for 24 h. The cells were exposed to hydrogen peroxide (1:50000 dilution) for 4 h and treated with the different formulations (50 and 5 µg/mL) or an aqueous solution of the extract. At the end of the experiment, the cells were washed with PBS, and the MTT assay was performed to evaluate the protective effect of the samples. Untreated cells (100% viability) were used as a negative control, whereas hydrogen peroxide-treated cells were used as a positive control.

### 2.7. Statistical Analysis of Data

The results were expressed as mean values ± standard deviations. Statistically significant differences were determined by using variance analysis, and the Student’s *t*-test. The minimum level of significance chosen was *p* < 0.05.

## 3. Results

### 3.1. Characterization of L. citriodora Extract

**Figure 1:**
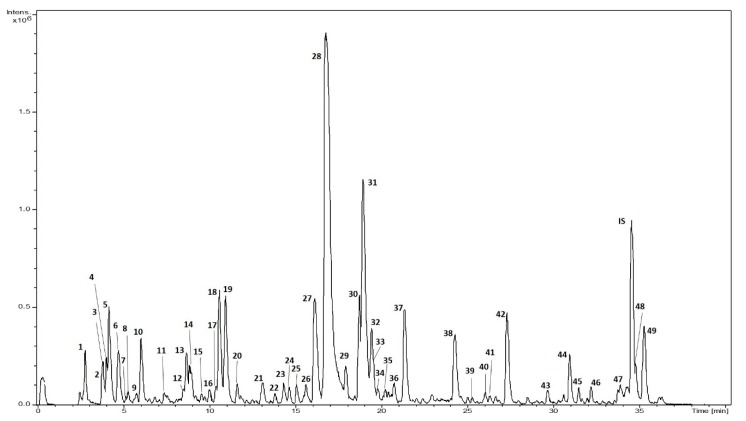
Base peak chromatogram of *L. citriodora* extract.

The extract from *L. citriodora* leaves was obtained by environmentally friendly microwave-assisted extraction, tuning the operating conditions to achieve the maximum concentration of polar compounds. The bioactive components of the extract were quantified by HPLC-ESI-TOF-MS (Figure 1). Table 2 shows a summary of the calculated parameters: retention time (min), m/z, molecular formula (M-H), chemical group, proposed name and amount expressed as mg of analyte per g of extract. The extract was found to be a complex mixture of polar compounds: 49 compounds were detected. Phenylpropanoids were the most abundant chemical group with 18 compounds accounting for 266 mg/g of extract. Among phenylpropanoids, verbascoside was the most representative (187 mg/g of extract), followed by isoverbascoside (57.3 mg/g of extract). Other phenylpropanoids, such as leucoseptoside A, verbascoside derivatives or cistanoside F, were found in relevant amounts. Oxoverbascoside, lariciresinol glucopyranoside, verbascoside A, lipedoside A I or osmanthisude were also detected, but in lower amounts. Flavonoids were the second major group found in the extract (18.2 mg/g of extract). Seven compounds were identified, with three aglycones and four glucuronic derivatives. Chrysoeriol-7-dilucuronide was the most abundant flavonoid (8.1 mg/g of extract). Luteolin-7-diglucuronide and apigenin-7-diglucuronide were detected in high amounts (3.81 and 2.6 mg/g of extract, respectively). Dimethyl quercetin was the most representative aglycone with 2.8 mg/g of extract, whereas methyl quercetin and dimethyl kaempferol were detected in lower amounts. Moreover, iridoid glycosides were a heterogeneous group with 13 compounds accounting for 12.9 mg/g of extract, with theveside, gardoside and ixoside being the most abundant compounds (> 1 mg/g of extract). The total polar content of the extract was almost 300 mg/g of extract.

Considering other studies on the recovery of phytochemicals from *L. citriodora* by pressurized liquid extraction (PLE), MAE was found to be an advanced extraction technology that allowed the recovery of a greater number of compounds from this botanical source. More specifically, three new compounds were found in our extract, as compared to the extracts obtained by PLE or conventional solid–liquid extraction [8,17].

Taking into account the findings of previous research, some phytochemicals in *L. citriodora* are responsible for the antioxidant effects of its extract, suggesting its use for the treatment of skin disorders associated with oxidative stress and inflammation [22,23,24].

### 3.2. Preparation and Characterization of Vesicles

The extract was incorporated in glycerosomes and PG-PEVs aiming at improving the stability of the phytochemicals extracted from *L. citriodora* and promoting their antioxidant efficacy. In order to preserve the green approach in the formulation project, the vesicles were prepared by using a simple preparation method that avoids the use of organic solvents. Only natural components and food-grade solvents were employed. In addition, the stability and carrier performances of the vesicles were improved by adding a biopolymer (biogelatin) extracted from fish by-product of *T. albacares*. *L. citriodora* extract loaded liposomes formulated without water-cosolvents and gelatin were prepared by the same procedure and used as a reference. The composition of the vesicles is reported in Table 1.

**Figure 2:**
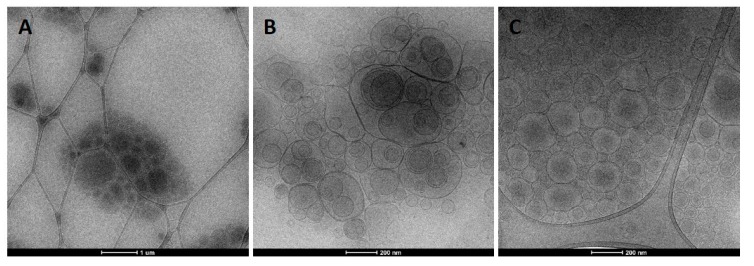
Cryo-TEM micrographs of *L. citriodora* extract loaded in liposomes (**A**), glycerosomes (**B**) and propylene glycol-containing vesicles (PG-PEVs; **C**).

The assessment of the morphology of the vesicles was achieved by cryo-TEM analyses. Liposomes (Figure 2A) were spherical and mainly unilamellar; bilamellar vesicles were also observed. Glycerosomes and PG-PEVs (Figure 2B,C, respectively) were multilamellar and multicompartment structures, probably due to the presence of glycols [25]. The effect of gelatin on the vesicle morphology was not relevant, as they showed a structure similar to the corresponding vesicles without gelatin (figures not shown).

The results of size, polydispersity index and zeta potential of the vesicles are reported in Table 3. Empty vesicles were also prepared, in order to evaluate the effect of the incorporation of the *L. citriodora* extract on the vesicles’ assembly. Empty liposomes were around 99 nm, and the loading of the extract led to an increase in mean diameter (151 nm, *p* < 0.01), which indicates an important contribution of the extract components to vesicle assembly. The addition of glycols to empty vesicles induced a slight increase in the mean diameter of glycerosomes (135 nm, *p* < 0.05 vs. liposomes) and a strong increase for PG-PEVs (310 nm). The addition of biogelatin caused a further increase in size for biogelatin-glycerosomes (202 nm, *p* < 0.05 vs. glycerosomes) and a decrease for biogelatin-PG-PEVs (235 nm). Unlike liposomes, the loading of the extract in the vesicles containing glycols caused a reduction in size, except for glycerosomes, which remained unchanged (133 nm, *p* > 0.05 vs. empty glycerosomes). The effect of the extract was especially evident in PG-PEVs whose size decreased from 310 to 109 nm, becoming the smallest vesicles (*p* < 0.05 vs. the other glycol-vesicles). This behavior can be ascribed to a positive contribution of propylene glycol to the intercalation and loading of the extract components within the vesicles. As reported in Table 2, the extract was mostly composed by phenylpropanoids and flavonoids with glucuronic moieties. These phenolic compounds had good solubility in water. Therefore, their localization within the vesicles might be in the internal polar regions of the bilayers, a fact that can cause the downsizing of the vesicles. The effect of biogelatin on the mean diameter of extract loaded glycol-vesicles was not relevant: it only caused a slight increase in size.

With regard to the polydispersity results, vesicles are considered monodispersed when PDI values approach zero, whereas values close to 1 indicate a heterogeneous size distribution [26]. The PDI values of our formulations displayed that the homogeneity of the vesicles loaded with *L. citriodora* extract was much higher than that of the corresponding empty particles, with the exception of liposomes. Extract-loaded glycerosomes and PG-PEVs were highly monodispersed (PDI < 0.2), much more than the corresponding empty vesicles (PDI up to 0.65). These results can be related to a possible interaction between glycerol or propylene glycol with the phenolic compounds of the extract, which positively affected the packing of the vesicles resulting in higher homogeneity. Furthermore, the biogelatin added to these formulations (5 mg/mL) could interact with the phospholipid providing, during vesicles’ production, a supramolecular structure where the biopolymer was intercalated in the bilayer, contributing to modify the lamellar arrangement and providing better results in terms of size and homogeneity (Table 3) [12]. Zeta potential was also measured, as it is predictive of the stability of colloidal dispersions [26]. The results (Table 3) showed that the incorporation of the extract modified the surface charge of the vesicles, with zeta potential values around −8 mV, regardless of the vesicle type.

Due to the great number of structurally different phytochemicals contained in the *L. citriodora* extract (Table 2), the entrapment efficiency was estimated by evaluating the antioxidant activity of the whole extract in dialyzed and non-dialyzed formulations. Liposomes showed the highest value of entrapment efficiency (89%). The presence of glycerol, propylene glycol or gelatin provided lower entrapment efficiency (65%). These results may be explained by taking into account the high solubility of extract phytochemicals in the solvent used for the purification of the formulations [1]. 

### 3.3. In Vitro Assays in Fibroblasts

#### 3.3.1. Biocompatibility Assay

**Figure 3:**
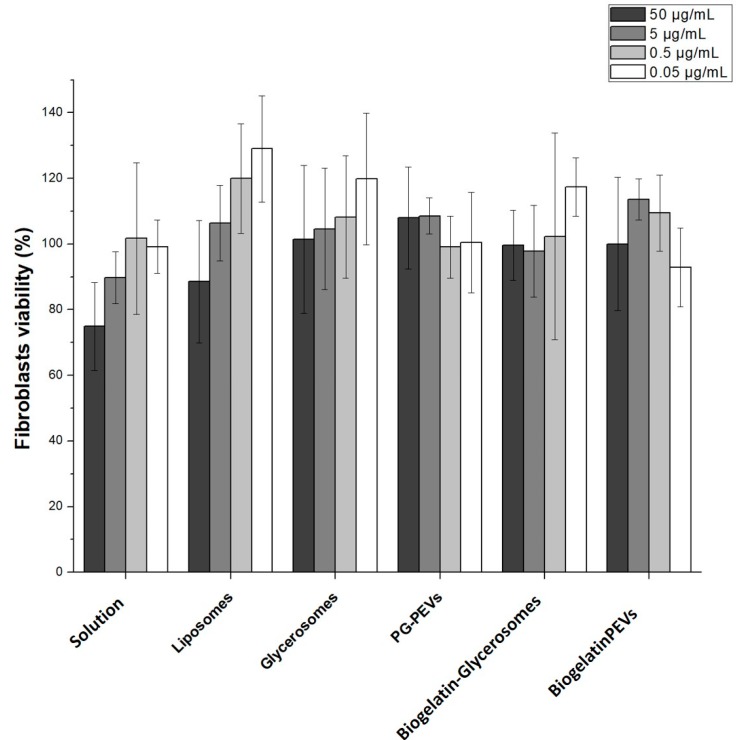
Biocompatibility assay of *L. citriodora* loaded vesicles performed on fibroblasts.

In order to evaluate the influence of the extract on the viability of skin cells, biocompatibility studies were performed by incubating 3T3 cells for 48 h with each formulation at different concentrations. As can be seen in Figure 3, when the higher concentrations (50 and 5 µg/mL) were used, the viability of the cells treated with the extract solution was lower than that detected in cells treated with the vesicle formulations. At lower concentrations (0.5 and 0.05 µg/mL) of the extract solution, the viability was 100%, which confirmed the biocompatibility of the extract. The incubation with extract-loaded vesicles did not alter the viability of the cells, which was always >100%, except when liposomes were used at the higher concentration (50 µg/mL). In the case of liposomes and glycerosomes, the viability was concentration-dependent: it increased as the concentration of the extract decreased. A different behavior was observed in cells treated with PG-PEVs: the viability was around 100% at all the concentrations tested. Opposing results were obtained when gelatin-glycol-vesicles were used. In the cells treated with gelatin-glycerosomes, the viability was around 100%, approaching 120% at the higher extract concentration. On the other hand, in the cells treated with PG-PEVs with gelatin, the higher viability (110%) was found at the intermediate concentrations (5 and 0.5 µg/mL). These findings point to the high biocompatibility of the extract-loaded vesicles, which even triggered a proliferative effect as a function of the concentration used. The statistical analysis has been included in Supplementary Material Table S1. 

#### 3.3.2. Antioxidant Assay

Given the promising physico-chemical characteristics of the vesicles (i.e., small size and homogeneity) and their high biocompatibility in skin cells (over 100% of fibroblast viability), further studies were carried out. In particular, the ability of the extract to protect skin cells from oxidative stress induced by hydrogen peroxide was evaluated. The effectiveness against oxidative stress was evaluated using vesicle formulations at 5 µg/mL because this concentration did not cause any toxic effect (viability ≥ 100%). In addition, the highest concentration (50 µg/mL) was used to evaluate if it could give different results. At this concentration, only extract-loaded liposomes caused a low toxicity, but a high extract concentration is expected to neutralize a greater number of the ROS produced by hydrogen peroxide (1:50000).

**Figure 4:**
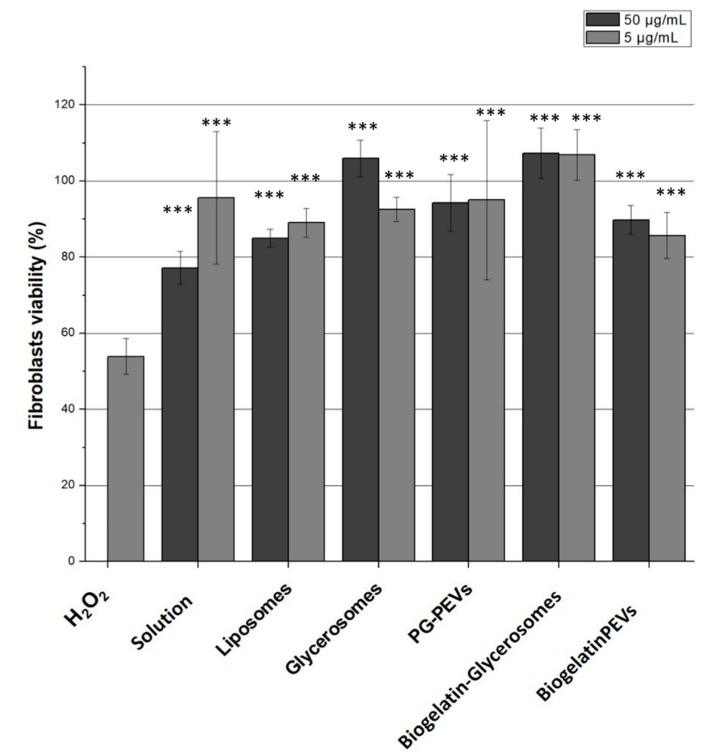
Antioxidant assay of *L. citriodora* loaded vesicles performed on fibroblasts (*** = *p* value < 0.001).

The exposure of fibroblasts to hydrogen peroxide increases the intracellular ROS concentration causing cell death via apoptosis or necrosis [27]. The results in Figure 4 confirmed a marked cell death after exposure to hydrogen peroxide, with an approximate 50% survival. The aqueous solution of the extract at 5 µg/mL was able to counteract the harmful effects of hydrogen peroxide, providing 95% of viability. The extract loaded vesicles provided a great protection against oxidative stress. The viability was always higher than 85%, with liposomes and biogelatin-PG-PEVs being the least effective, and glycerosomes and biogelatin-glycerosomes being the most effective (> 100% viability). Although fibroblasts are located in the deep strata of the skin, they can be reached and protected by the bioactive compounds of the extract thanks to the enhanced delivery of the vesicles [1]. Overall, all formulations demonstrated a significant potential in protecting skin cells from damages caused by H_2_O_2_. Nevertheless, glycerosomes and biogelatin-glycerosomes displayed the best results in terms of in vitro biocompatibility and antioxidant protective effect, which suggests a better ability to interact with cells and promote the release of the extract bioactives.

## 4. Conclusions

The performed environmentally friendly microwave-assisted extraction allowed the production of an extract from *L. citriodora* rich in polyphenolic compounds. The obtained extract showed a good protective effect against oxidative stress in skin cells. The total-green approach allowed the development of phospholipid vesicles based on biocompatible components. The vesicle formulations enhanced the ability of the extract to neutralize the ROS produced by hydrogen peroxide in skin cells. Particularly, glycerosomes and biogelatin-glycerosomes showed the highest protective effects. These promising results suggest the use of *L. citriodora* extract-loaded biogelatin-glycerosomes for the production of innovative cosmeceuticals as a green alternative to ameliorate skin disorders.

## Figures and Tables

**Table 1:** Composition of the vesicle formulations.

Formulation	*L. citriodora* Extract (mg)	P90G (mg)	Gelatine (mg)	H_2_O (mL)	Glycerol (mL)	Propylene Glycol (mL)
Empty liposomes	-	180	-	1	-	-
Empty glycerosomes	-	180	-	0.75	0.25	-
Empty PG-PEVs	-	180	-	0.75	-	0.25
Empty biogelatin-glycerosomes	-	180	5	0.75	0.25	-
Empty biogelatin-PG-PEVs	-	180	5	0.75	-	0.25
*L. citriodora* liposomes	50	180	-	1	-	-
*L. citriodora* glycerosomes	50	180	-	0.75	0.25	-
*L. citriodora* PG-PEVs	50	180	-	0.75	-	0.25
*L. citriodora* biogelatin-glycerosomes	50	180	5	0.75	0.25	-
*L. citriodora* biogelatin-PG-PEVs	50	180	5	0.75	-	0.25

**Table 2:** Qualitative and quantitative results of *L. citriodora* extract.

Peak	RT (min)	m/z Cal	m/z Exp	Formula (M-H)	Proposed Compound	Chemical Group	Quantification mg Analyte/g Extract
**1**	2.8	195.0510	195.0508	C_6_H_11_O_7_	Gluconic acid	Organic acid	-
**2**	3.8	373.1140	373.1131	C_16_H_21_O_10_	Gardoside	Iridoid glycoside	4.4 ± 0.3
**3**	3.9	391.1246	391.1241	C_16_H_23_O_11_	Shanzhiside	Iridoid glycoside	0.24 ± 0.02
**4**	4.0	387.0933	387.0933	C_16_H_19_O_11_	Ixoside	Iridoid glycoside	1.78 ± 0.03
**5**	4.2	461.1664	461.1670	C_20_H_29_O_12_	Verbasoside	Phenylpropanoid	3.56 ± 0.02
**6**	4.8	487.1457	487.1443	C_21_H_27_O_13_	Cistanoside F	Phenylpropanoid	1.72 ± 0.06
**7**	5.2	203.0925	203.0907	C_9_H_15_O_5_	UK 1	-	-
**8**	5.3	475.1398	475.1435	C_20_H_27_O_13_	Primeverin	Other	-
**9**	5.8	285.0616	285.0591	C_12_H_13_O_8_	Pyrocatechol Glucuronide	Other	-
**10**	5.9	389.1089	389.1077	C_16_H_21_O_11_	Theveside	Iridoid glycoside	4.8 ± 0.1
**11**	7.4	449.1301	449.1301	C_18_H_25_O_13_	Myxopyroside	Iridoid glycoside	0.271 ± 0.005
**12**	8.5	489.1614	489.1618	C_21_H_29_O_13_	Teucardoside	Iridoid glycoside	0.108 ± 0.002
**13**	8.7	387.1661	387.1640	C_18_H_27_O_9_	Tuberonic acid glucoside	Other	-
**14**	9.0	433.2079	433.2077	C_20_H_33_O_10_	UK 2	-	-
**15**	9.5	641.2087	641.2028	C_29_H_37_O_16_	β-Hydroxyverbascoside derivative	Phenylpropanoid	0.28 ± 0.03
**16**	10.0	641.2087	641.2065	C_29_H_37_O_16_	β-Hydroxyisoverbascoside derivative	Phenylpropanoid	0.65 ± 0.05
**17**	10.6	639.1931	639.1912	C_29_H_35_O_16_	β-Hydroxyverbascoside	Phenylpropanoid	1.85 ± 0.09
**18**	10.6	637.1046	637.1048	C_27_H_25_O_18_	Luteolin-7-diglucoronide	Flavonoid	3.81 ± 0.01
**19**	11.0	639.1931	639.1923	C_29_H_35_O_16_	β-Hydroxyisoverbascoside	Phenylpropanoid	1.93 ± 0.04
**20**	11.6	553.1563	553.1572	C_25_H_29_O_14_	Lippioside II	Iridoid glycoside	0.21 ± 0.03
**21**	13.1	639.1872	639.1879	C_36_H_31_O_11_	UK 3	-	-
**22**	13.8	637.1774	637.1811	C_29_H_33_O_16_	Oxoverbascoside	Phenylpropanoid	0.043 ± 0.003
**23**	14.3	621.1097	621.1115	C_27_H_25_O_17_	Apigenin-7-diglucoronide	Flavonoid	0.39 ± 0.01
**24**	14.6	535.1457	535.1442	C_25_H_27_O_13_	Lippioside I derivative	Iridoid glycoside	0.11 ± 0.01
**25**	15.1	537.1614	537.1595	C_25_H_29_O_13_	Lippioside I	Iridoid glycoside	0.35 ± 0.01
**26**	15.6	653.2087	653.2058	C_30_H_37_O_16_	Campneoside I	Phenylpropanoid	NQ
**27**	16.1	651.1355	651.1212	C_28_H_27_O_18_	Chrysoeriol-7-diglucuronide	Flavonoid	8.1 ± 0.7
**28**	16.4	623.1981	623.1998	C_29_H_35_O_15_	Verbascoside	Phenylpropanoid	187 ± 2
**29**	17.9	521.2028	521.2031	C_26_H_33_O_11_	Lariciresinol glucopyranoside	Phenylpropanoid	1.08 ± 0.04
**30**	18.7	667.2244	667.2230	C_31_H_39_O_16_	Verbascoside A	Phenylpropanoid	0.62 ± 0.01
**31**	18.9	623.1981	623.1991	C_29_H_35_O_15_	Isoverbascoside	Phenylpropanoid	57.3 ± 0.8
**32**	19.4	623.1981	623.1973	C_29_H_35_O_15_	Forsythoside A	Phenylpropanoid	0.39 ± 0.03
**33**	19.4	549.1614	549.1645	C_26_H_29_O_13_	Lippianoside B	Iridoid glycoside	0.18 ± 0.06
**34**	19.8	521.1664	521.1673	C_25_H_29_O_12_	Hydroxycampsiside	Iridoid glycoside	0.16 ± 0.02
**35**	20.2	607.2032	607.2057	C_29_H_35_O_14_	Lipedoside A I	Phenylpropanoid	0.13 ± 0.03
**36**	20.7	551.1770	551.1774	C_26_H_31_O_13_	Durantoside I	Iridoid glycoside	0.32 ± 0.01
**37**	21.3	637.2138	637.2150	C_30_H_37_O_15_	Leucoseptoside A	Phenylpropanoid	4.07 ± 0.01
**38**	24.2	635.1254	635.1277	C_28_H_27_O_17_	Acacetin-7-diglucoronide	Flavonoid	2.6 ± 0.1
**39**	25.2	551.2498	551.2539	C_28_H_39_O_11_	UK 4	-	-
**40**	26.0	467.2134	467.2146	C_20_H_35_O_12_	UK 5	-	-
**41**	26.3	549.1614	549.1640	C_26_H_29_O_13_	UK 6	-	-
**42**	27.2	651.2294	651.2303	C_31_H_39_O_15_	Martynoside or isomer	Phenylpropanoid	3.0 ± 0.1
**43**	29.6	651.2294	651.2293	C_31_H_39_O_15_	Martynoside or isomer	Phenylpropanoid	0.69 ± 0.02
**44**	30.9	591.2083	591.2163	C_29_H_35_O_13_	Osmanthuside B	Phenylpropanoid	1.33 ± 0.03
**45**	31.4	569.2240	569.2243	C_27_H_37_O_13_	Manuleoside H	Iridoid glycoside	NQ
**46**	32.1	315.0510	315.0507	C_16_H_11_O_7_	Methyl quercetin	Flavonoid	NQ
**47**	33.8	327.2177	327.2182	C_18_H_31_O_5_	UK 7	-	-
**48**	34.7	299.0561	299.0575	C_16_H_11_O_6_	Dimethyl kaemferol	Flavonoid	0.55 ± 0.03
**49**	35.2	329.0667	329.0669	C_17_H_13_O_7_	Dimethyl quercetin	Flavonoid	2.8 ± 0.1

NQ: not quantified: compound detected but with a concentration between the detection and quantification limits. UK: unknown compound, compound that was not able to identify.

**Table 3:** Characterization of the vesicles including mean diameter (MD), polydispersity index (PDI), zeta potential (ZP) and entrapment efficiency (EE). Mean values ± standard deviations were obtained from at least 6 replicates.

Formulation	MD (nm)	PDI	ZP (mV)	EE (%)
Empty liposomes	99 ± 8	0.199	−12 ± 1	
Empty glycerosomes	135 ± 3	0.265	−18 ± 2	
Empty PG-PEVs	310 ± 4	0.652	−21 ± 1	
Empty biogelatin-glycerosomes	202 ± 12	0.456	−3 ± 1	
Empty biogelatin-PG-PEVs	235 ± 7	0.577	−2 ± 1	
*L. citriodora* liposomes	151 ± 13	0.284	−7 ± 1	89 ± 7
*L. citriodora* glycerosomes	133 ± 9	0.157	−8 ± 2	66 ± 3
*L. citriodora* PG-PEVs	109 ± 4	0.191	−7 ± 2	65 ± 4
*L. citriodora* biogelatin-glycerosomes	149 ± 1	0.117	−8 ± 1	63 ± 8
*L. citriodora* biogelatin-PG-PEVs	134 ± 5	0.237	−8 ± 1	63 ± 15

PG: propylene glycol.

## Supplementary Materials

The following are available online at https://www.mdpi.com/2079-4991/10/4/765/s1, Table S1: ANOVA of results obtained in biocompatibility assays.

## References

[1] Manconi M., Manca M.L., Marongiu F., Caddeo C., Castangia I., Petretto G.L., Pintore G., Sarais G., D’hallewin G., Zaru M. (2016). Chemical characterization of Citrus limon var. pompia and incorporation in phospholipid vesicles for skin delivery. Int. J. Pharm..

[2] Leyva-Jiménez F.J., Lozano-Sánchez J., de la Cádiz-Gurrea M.L., Arráez-Román D., Segura-Carretero A. (2019). Functional Ingredients based on Nutritional Phenolics. A Case Study against Inflammation: Lippia Genus. Nutrients.

[3] Liguori I., Russo G., Curcio F., Bulli G., Aran L., Della-Morte D., Gargiulo G., Testa G., Cacciatore F., Bonaduce D. (2018). Oxidative stress, aging, and diseases. Clin. Interv. Aging.

[4] Sukadeetad K., Nakbanpote W., Heinrich M., Nuengchamnong N. (2018). Effect of drying methods and solvent extraction on the phenolic compounds of Gynura pseudochina (L.) DC. leaf extracts and their anti-psoriatic property. Ind. Crops Prod..

[5] Fu R., Zhang Y., Peng T., Guo Y., Chen F. (2015). Phenolic composition and effects on allergic contact dermatitis of phenolic extracts Sapium sebiferum (L.) Roxb. leaves. J. Ethnopharmacol..

[6] de Melo M.N.O., Oliveira A.P., Wiecikowski A.F., Carvalho R.S., de Castro J.L., de Oliveira F.A.G., Pereira H.M.G., da Veiga V.F., Capella M.M.A., Rocha L. (2018). Phenolic compounds from Viscum album tinctures enhanced antitumor activity in melanoma murine cancer cells. Saudi Pharm. J..

[7] Argyropoulou C., Daferera D., Tarantilis P.A., Fasseas C., Polissiou M. (2007). Chemical composition of the essential oil from leaves of Lippia citriodora H.B.K. (Verbenaceae) at two developmental stages. Biochem. Syst. Ecol..

[8] Leyva-Jiménez F.J., Lozano-Sánchez J., Borrás-Linares I., Arráez-Román D., Segura-Carretero A. (2018). Comparative study of conventional and pressurized liquid extraction for recovering bioactive compounds from Lippia citriodora leaves. Food Res. Int..

[9] de la Cádiz-Gurrea M.L., Micol V., Joven J., Segura-Carretero A., Fernández-Arroyo S. (2018). Different behavior of polyphenols in energy metabolism of lipopolysaccharide-stimulated cells. Food Res. Int..

[10] Quirantes-Piné R., Arráez-Román D., Segura-Carretero A., Fernández-Gutiérrez A. (2010). Characterization of phenolic and other polar compounds in a lemon verbena extract by capillary electrophoresis-electrospray ionization-mass spectrometry. J. Sep. Sci..

[11] Kitagawa S., Tanaka Y., Tanaka M., Endo K., Yoshii A. (2009). Enhanced skin delivery of quercetin by microemulsion. J. Pharm. Pharmacol..

[12] Castangia I., Caddeo C., Manca M.L., Casu L., Latorre A.C., Díez-Sales O., Ruiz-Saurí A., Bacchetta G., Fadda A.M., Manconi M. (2015). Delivery of liquorice extract by liposomes and hyalurosomes to protect the skin against oxidative stress injuries. Carbohydr. Polym..

[13] Bucci P., Prieto M.J., Milla L., Calienni M.N., Martinez L., Rivarola V., Alonso S., Montanari J. (2018). Skin penetration and UV-damage prevention by nanoberries. J. Cosmet. Dermatol..

[14] Ganesan P., Choi D.K. (2016). Current application of phytocompound-based nanocosmeceuticals for beauty and skin therapy. Int. J. Nanomed..

[15] Manca M.L., Jos J., Peris J.E., Melis V., Valenti D., Cardia M.C., Lattuada D., Escribano-Ferrer E., Fadda A.M., Manconi M. (2015). Nanoincorporation of curcumin in polymer-glycerosomes and evaluation of their in vitro-in vivo suitability as pulmonary delivery systems. RSC Adv..

[16] Bonechi C., Donati A., Tamasi G., Leone G., Consumi M., Rossi C., Lamponi S., Magnani A. (2018). Protective effect of quercetin and rutin encapsulated liposomes on induced oxidative stress. Biophys. Chem..

[17] Păvăloiu R.-D., Sha’at F., Bubueanu C., Deaconu M., Neagu G., Sha’at M., Anastasescu M., Mihailescu M., Matei C., Nechifor G. (2019). Polyphenolic Extract from Sambucus ebulus L. Leaves Free and Loaded into Lipid Vesicles. Nanomaterials.

[18] Sousa S., Vázquez J., Pérez-Martín R., Carvalho A., Gomes A. (2017). Valorization of By-Products from Commercial Fish Species: Extraction and Chemical Properties of Skin Gelatins. Molecules.

[19] Leyva-Jiménez F.J., Lozano-Sánchez J., Borrás-Linares I., Arráez-Román D., Segura-Carretero A. (2019). Manufacturing design to improve the attainment of functional ingredients from Aloysia citriodora leaves by advanced microwave technology. J. Ind. Eng. Chem..

[20] Manca M.L., Castangia I., Matricardi P., Lampis S., Fernàndez-Busquets X., Fadda A.M., Manconi M. (2014). Molecular arrangements and interconnected bilayer formation induced by alcohol or polyalcohol in phospholipid vesicles. Colloids Surf. B Biointerfaces.

[21] ISO (2009). ISO 10993-5: Biological Evaluation of Medical Devices-Part 5: Test for In Vitro Cytotoxicity.

[22] de la Cádiz-Gurrea M.L., Olivares-Vicente M., Herranz-López M., Román-Arráez D., Fernández-Arroyo S., Micol V., Segura-Carretero A. (2018). Bioassay-guided purification of Lippia citriodora polyphenols with AMPK modulatory activity. J. Funct. Foods.

[23] Deepak M., Handa S.S. (2000). Antiinflammatory activity and chemical composition of extracts of Verbena officinalis. Phytother. Res..

[24] Carrera-Quintanar L., Funes L., Viudes E., Tur J., Micol V., Roche E., Pons A. (2012). Antioxidant effect of lemon verbena extracts in lymphocytes of university students performing aerobic training program. Scand. J. Med. Sci. Sports.

[25] Manconi M., Petretto G., D’hallewin G., Escribano E., Milia E., Pinna R., Palmieri A., Firoznezhad M., Peris J.E., Usach I. (2018). Thymus essential oil extraction, characterization and incorporation in phospholipid vesicles for the antioxidant/antibacterial treatment of oral cavity diseases. Colloids Surf. B Biointerfaces.

[26] Saber F.R., Abdelbary G.A., Salama M.M., Saleh D.O., Fathy M.M., Soliman F.M. (2018). UPLC/QTOF/MS profiling of two Psidium species and the in-vivo hepatoprotective activity of their nano-formulated liposomes. Food Res. Int..

[27] Liu C., Guo H., DaSilva N.A., Li D., Zhang K., Wan Y., Gao X.-H., Chen H.-D., Seeram N.P., Ma H. (2019). Pomegranate (Punica granatum) phenolics ameliorate hydrogen peroxide-induced oxidative stress and cytotoxicity in human keratinocytes. J. Funct. Foods.

