# Supplementary material for: Incorporation of Lippia citriodora Microwave Extract into Total-Green Biogelatin-Phospholipid Vesicles to Improve Its Antioxidant Activity
Source: Nanomaterials (Basel). 2020 Apr 16;10(4):765. doi: 10.3390/nano10040765 (PMC7221744; doi:10.3390/nano10040765)
Supplement: Supplementary file 1 [file nanomaterials-10-00765-s001.pdf]

nanomaterials

Type of the Paper (Article)

Incorporation of *Lippia Citriodora* Microwave Extract into Total-green Biogelatin-phospholipid Vesicles to Improve Its Antioxidant Activity

Francisco Javier Leyva-Jiménez [1], Maria Letizia Manca [2], Maria Manconi [2], Carla Caddeo [2], José Antonio Vázquez [3], Jesús Lozano-Sánchez [1,4,*], Elvira Escribano-Ferrer [5,6], David Arráez-Román [1,7] and Antonio Segura-Carretero [1,7]

[1] Functional Food Research and Development Center, Health Science Technological Park, Avenida del Conocimiento s/n, E-18016 Granada, Spain; jleyva@cidaf.es (F.J.L.-J.); jesusls@ugr.es (J. L.-S.); darraez@ugr.es (D.A.-R.); ansegura@ugr.es (A.S.-C.).

[2] Department of Scienze della Vita e dell'Ambiente, University of Cagliari, via Ospedale 72, 09124 Cagliari, Italy; mlmanca@unica.it (M.L.M.); manconi@unica.it (M.M.); caddeoc@unica.it (C.C.).

[3] Group of Recycling and Valorization of Waste Materials (REVAL), Marine Research Institute (IIM-CSIC), C/Eduardo Cabello, 6, CP36208, Vigo, Spain; jvazquez@iim.csic.es (J.A.V.)

[4] Department of Food Science and Nutrition, University of Granada, Campus of Cartuja, 18011 Granada, Spain; jesusls@ugr.es (J. L.-S.).

[5] Biopharmaceutics and Pharmacokinetics Unit, Institute for Nanoscience and Nanotechnology, University of Barcelona, 08193 Barcelona, Spain; eescribano@ub.edu (E.E.-F.)

[6] CIBER Physiopathology of Obesity and Nutrition (CIBEROBN), Institute of Health Carlos III, 28029 Madrid. Spain; eescribano@ub.edu (E.E.-F.)

[7] Department of Analytical Chemistry, Faculty of Sciences, University of Granada, Fuentenueva s/n, E-18071 Granada, Spain; darraez@ugr.es (D.A.-R.); ansegura@ugr.es (A.S.-C.).

* Correspondence: jesusls@ugr.es; Tel.: +34-958-637083

nanomaterials

MDPI

Supplementary material Table S1: ANOVA of results obtained in biocompatibility assays.

Dependent variable	Formulation	Formulation	Significance
50 μg/mL	Solution	Liposomes	0
		Glycerosomes	0
		PG-PEVS	1
		Glycerosomes+biogelatin	0
		PG-PEVS+biogelatin	0
	Liposomes	Solution	0
		Glycerosomes	0
		PG-PEVS	0
		Glycerosomes+biogelatin	0
		PG-PEVS+biogelatin	0
	Glycerosomes	Solution	0
		Liposomes	0
		PG-PEVS	0
		Glycerosomes+biogelatin	0
		PG-PEVS+biogelatin	0
	PG-PEVS	Solution	1
		Liposomes	0
		Glycerosomes	0
		Glycerosomes+biogelatin	0
		PG-PEVS+biogelatin	0
	Glycerosomes+biogelatin	Solution	0
		Liposomes	0
		Glycerosomes	0
		PG-PEVS	0
		PG-PEVS+biogelatin	0
	PG-PEVS+biogelatin	Solution	0
		Liposomes	0
		Glycerosomes	0
		PG-PEVS	0
		Glycerosomes+biogelatin	0
5 μg/mL	Solution	Liposomes	0
		Glycerosomes	0
		PG-PEVS	0
		Glycerosomes+biogelatin	0

nanomaterials

		PG-PEVS+biogelatin	0
	Liposomes	Solution	0
		Glycerosomes	0
		PG-PEVS	0
		Glycerosomes+biogelatin	0
		PG-PEVS+biogelatin	0
	Glycerosomes	Solution	0
		Liposomes	0
		PG-PEVS	0
		Glycerosomes+biogelatin	0
		PG-PEVS+biogelatin	0
	PG-PEVS	Solution	0
		Liposomes	0
		Glycerosomes	0
		Glycerosomes+biogelatin	0
		PG-PEVS+biogelatin	0
	Glycerosomes+biogelatin	Solution	0
		Liposomes	0
		Glycerosomes	0
		PG-PEVS	0
		PG-PEVS+biogelatin	0
	PG-PEVS+biogelatin	Solution	0
		Liposomes	0
		Glycerosomes	0
		PG-PEVS	0
		Glycerosomes+biogelatin	0
**0.5 µg/mL**	Solution	Liposomes	0
		Glycerosomes	0
		PG-PEVS	0
		Glycerosomes+biogelatin	0
		PG-PEVS+biogelatin	0
	Liposomes	Solution	0
		Glycerosomes	0
		PG-PEVS	0
		Glycerosomes+biogelatin	0
		PG-PEVS+biogelatin	0
	Glycerosomes	Solution	0

nanomaterials

		Liposomes	0
		PG-PEVS	0
		Glycerosomes+biogelatin	0
		PG-PEVS+biogelatin	0
	PG-PEVS	Solution	0
		Liposomes	0
		Glycerosomes	0
		Glycerosomes+biogelatin	0
		PG-PEVS+biogelatin	0
	Glycerosomes+biogelatin	Solution	0
		Liposomes	0
		Glycerosomes	0
		PG-PEVS	0
		PG-PEVS+biogelatin	0
	PG-PEVS+biogelatin	Solution	0
		Liposomes	0
		Glycerosomes	0
		PG-PEVS	0
		Glycerosomes+biogelatin	0
**0.05 µg/mL**	Solution	Liposomes	1
		Glycerosomes	0
		PG-PEVS	0
		Glycerosomes+biogelatin	0
		PG-PEVS+biogelatin	0
	Liposomes	Solution	1
		Glycerosomes	0
		PG-PEVS	1
		Glycerosomes+biogelatin	0
		PG-PEVS+biogelatin	1
	Glycerosomes	Solution	0
		Liposomes	0
		PG-PEVS	1
		Glycerosomes+biogelatin	0
		PG-PEVS+biogelatin	1
	PG-PEVS	Solution	0
		Liposomes	1
		Glycerosomes	1

nanomaterials

		Glycerosomes+biogelatin	1
		PG-PEVS+biogelatin	0
	Glycerosomes+biogelatin	Solution	0
		Liposomes	0
		Glycerosomes	0
		PG-PEVS	1
		PG-PEVS+biogelatin	1
	PG-PEVS+biogelatin	Solution	0
		Liposomes	1
		Glycerosomes	1
		PG-PEVS	0
		Glycerosomes+biogelatin	1

1 indicates a significative difference (p<0.05); 0 indicates a non-significative difference (p>0.05).
